# Patient-reported propagated sensation along meridians during acupuncture for lumbar disc herniation: protocol for an exploratory prospective cohort study with multimodal neurophysiological and immune measurements

**DOI:** 10.3389/fneur.2026.1935793

**Published:** 2026-10-07

**Authors:** Jianhao Huang, Yi Chen, Weiling Zheng, Xinjing Yang, Xiaopian Guo, Dawei Si, Yufeng Xie

**Affiliations:** 1 Shenzhen Hospital (Futian) of Guangzhou University of Chinese Medicine, Shenzhen, China; 2 Macao Polytechnic University, Macau, China; 3 Shenzhen University, Shenzhen, China

**Keywords:** acupuncture, clinical outcomes, lumbar disc herniation, multimodal biosignals, physiological correlates, propagated sensation along meridians, prospective cohort study

## Abstract

**Background:**

Propagated sensation along meridians (PSM) refers to the radiation of needling sensation beyond the needling site along meridian pathways. In traditional acupuncture theory, PSM has long been considered an important factor associated with clinical efficacy, particularly in conditions like lumbar disc herniation where pain radiates along the lower limb. However, this phenomenon has not been well characterized by modern neurobiological methods. Whether patients who report PSM show distinct autonomic, sensory, or motor responses, and whether this relates to better outcomes, remains unclear. In this study, we aim to explore the clinical and physiological correlates of patient-reported PSM in a cohort of LDH patients receiving standardized acupuncture.

**Methods and analysis:**

This single-center, prospective, observational cohort study will enroll 204 LDH patients with radicular pain. Participants will be classified into three groups based on PSM reporting across three treatment sessions corroborated by assessor-documented needle-evoked muscle twitches: PSM-positive, PSM-negative, and indeterminate. The primary endpoint is the clinical improvement rate based on Japanese Orthopaedic Association (JOA) scores. Secondary endpoints include pain intensity (VAS), functional status (JOA), multimodal neurophysiological parameters (EEG, sEMG, EDA, EOG), serological cytokine profiles (IL-1*β*, IL-2, IL-4, IL-5, IL-6, IL-8, IL-10, IL-12p70, IL-17, IFN-γ, TNF-*α*, and IFN-*α*), and the Massachusetts General Hospital Acupuncture Sensation Scale (MASS) administered post-session to quantify PSM intensity and spreading. Data will be analyzed using linear mixed-effects models, with results reported as effect estimates with 95% confidence intervals and false discovery rate correction within signal families; exploratory multimodal analyses will be used to identify physiological features associated with PSM.

**Expected results:**

We hypothesize that the PSM-positive cohort will show greater clinical improvement than the PSM-negative cohort, with larger reductions in radicular pain and better functional recovery following standardized acupuncture.

**Clinical trial registration:**

ITMCTR2025001534, https://itmctr.ccebtcm.org.cn.

## 1 Introduction

Propagated Sensation along Meridians (PSM) describes the phenomenon in which needling-evoked sensation radiates beyond the insertion site along meridian pathways (1, 2). Classical acupuncture texts describe this as “Qi arrival” at the disease location (3), and PSM has long been regarded as a favorable prognostic sign in traditional acupuncture practice; whether patient-reported PSM is associated with clinical outcomes in a measurable way, however, remains empirically unclear.

Traditional acupuncture distinguishes two related but distinct sensory phenomena. Deqi refers to a composite sensory experience characterized by the practitioner’s perception of needle grasp (sinking and tightness) (4) and the patient’s localized sensations of soreness, numbness, distension, or heaviness (5). PSM, by contrast, emphasizes directional propagation of sensation along meridian pathways.

The Miraculous Pivot (Ling Shu) establishes the foundational principle that “the essence of needling lies in the arrival of Qi, after which efficacy follows” (3). Historical accounts in the Records of the Three Kingdoms describe Hua Tuo’s deliberate guidance of needle sensation toward the affected site (6). The Ode to the Elucidation of the Mystery (Biao You Fu) further emphasizes a temporal aspect, asserting that rapid Qi arrival correlates with swift therapeutic response while delayed arrival indicates poorer prognosis (7).

Whether PSM reflects a genuine biological process or is purely subjective has long been debated. More than three decades of basic research, led primarily by the Fujian Academy of Chinese Medical Sciences, has produced converging, though largely indirect, evidence of measurable physiological correlates. Large-scale surveys across China found that PSM occurs frequently in the general population and can be regarded as a normal physiological phenomenon (8). Infrared thermal imaging further showed that, without external stimulation, the body surface spontaneously exhibits radiant tracks along meridian courses (IRRTM) that align closely with the classical fourteen-meridian pathways; these tracks are dynamically regulated and often bilateral (9), and correspond to tissues with higher microcirculatory perfusion, oxygen partial pressure, local temperature, and energy metabolism than adjacent non-meridian regions (10–13). Heating meridian points triggers a bidirectional, long-distance thermal response confined to the meridian course, whereas heating comparable non-meridian points does not, indicating directionally organized thermal conductivity in meridian-associated tissues (14); needling can likewise induce or regularize the corresponding IRRTM (15).

At the central nervous system level, topographical mapping of cortical somatosensory evoked potentials during genuine versus experimentally simulated PSM indicated that activation of a peripheral process, rather than imagination, contributes substantially to the phenomenon, consistent with a peripheral-central integration hypothesis (16). Subsequent neuroelectrophysiological recordings reported impulses propagating along the meridian course during PSM (1), and gene expression profiling has identified molecular signatures of the PSM state (17). Ongoing NSFC-funded work is applying artificial intelligence-assisted infrared image recognition to characterize the objective manifestations of PSM (8). Together, these findings indicate that PSM is a biologically plausible phenomenon with measurable peripheral and central correlates, motivating the multimodal neurophysiological and immune measurements in the present protocol.

Despite this biological plausibility, studying PSM faces a practical challenge: its defining feature, dynamic propagation along defined meridian pathways, cannot be captured by existing measurement tools, and in current clinical practice PSM identification relies predominantly on patients’ verbal reports. Instruments such as the Visual Analogue Scale (VAS) and the Massachusetts General Hospital Acupuncture Sensation Scale (MASS) have been validated for quantifying general Deqi sensations, including intensity and spreading (7, 18). Moreover, needling also evokes objectively observable motor phenomena. Needle-evoked involuntary muscle twitches, known as “Zha-Tiao” (twitch-eliciting needling) in acupuncture practice, are regarded as a principal objective manifestation of Deqi beyond subjective needle sensations, and are elicitable through stimulation of intramuscular targets such as muscle spindles, motor point areas, and neurovascular hilums (19). Such local twitch responses can be documented electromyographically and ultrasonographically, and their elicitation has been associated with greater pain relief in trigger-point needling (19, 20). Notably, needling of hyperexcitable acu-reflex points may evoke propagated needle sensations accompanied by overt twitch responses (19), and, in our clinical experience, patients reporting PSM during acupuncture for lumbocrural disorders frequently exhibit involuntary twitches of the lower-limb musculature. Accordingly, in this protocol PSM will be characterized by combining patients’ subjective reports with assessor-documented, sEMG-recordable needle-evoked lower-limb muscle twitches, so that PSM classification rests on both subjective and objective components rather than on a verbal label alone (see Methods, Sections 2.4 and 2.5.4). To date, however, no study has systematically examined whether PSM characterized in this manner is associated with clinical and physiological outcomes in a standardized acupuncture setting. Key constructs are operationally defined in Table 1.

**Table 1:** Operational definitions of key constructs.

Term	Operational definition in this study
Deqi	The composite of local needling sensations (soreness, numbness, distension, heaviness) perceived at the needled site
PSM	The propagation of needling sensations along meridian pathways beyond the needled site, as reported by the patient
PSM-positive cohort	Participants reporting PSM in ≥2 out of 3 treatment sessions with at least one assessor-documented needle-evoked lower-limb muscle twitch
PSM-negative cohort	PSM reported in 0 out of 3 treatment sessions and no needle-evoked lower-limb twitch documented
Indeterminate group	PSM reported in exactly 1 out of 3 treatment sessions, or discordance between subjective reports and twitch documentation

Lumbar disc herniation (LDH) provides a well-defined clinical and pathological context to systematically investigate these questions and address the current methodological gaps. As the predominant etiology of lumbosacral radicular pain, LDH imposes a substantial burden on global healthcare systems due to its high prevalence and associated disability rates (21). Among conservative management strategies, acupuncture has been recommended as a nonpharmacological therapy in several clinical guidelines (22, 23), with accumulating clinical and mechanistic evidence further supporting its use in this condition (24, 25). However, the specific contribution of PSM to these established therapeutic outcomes remains insufficiently characterized.

Patients with LDH-related radicular pain provide a relevant model for studying PSM, as the characteristic symptom of radiating pain along the lower limb shares a similar spatial distribution with the propagated sensation elicited by acupuncture. This symptomatic overlap, however, also presents a methodological challenge. Distinguishing PSM from pre-existing or fluctuating neurological symptoms requires temporally anchored assessment relative to needle manipulation and systematic baseline symptom recording, complemented by the concurrent documentation of needle-evoked lower-limb twitches as an objectively observable anchor (see Methods, Section 2.5.4). At the same time, this overlap provides an opportunity to explore whether acupuncture-induced sensation propagation along the same pathway is associated with improved therapeutic outcomes, beyond what can be explained by localized needle sensation alone. Moreover, LDH involves both peripheral nerve inflammation (26) and altered spinal nociceptive processing (27). This pathophysiological context offers a useful entry point for exploring whether the dynamic propagation of PSM correlates with changes in the neuro-immune microenvironment. Based on this rationale, we will conduct a prospective cohort study in patients with LDH-related radicular pain.

Patients will be assigned to PSM-positive, PSM-negative, or indeterminate groups based on their report of propagated sensation across three standardized treatment sessions, together with assessor-documented needle-evoked lower-limb muscle twitches (see Methods, Section 2.4). We will compare radicular pain intensity (VAS) and functional disability (JOA) scores across groups to examine whether PSM reporting is associated with differential clinical outcomes.

In parallel, we will record continuous multichannel EEG, sEMG, EOG, and EDA throughout treatment sessions and collect serial blood samples for cytokine profiling, to explore whether neurophysiological and inflammatory patterns differ between patients who report PSM and those who do not; an exploratory machine learning approach will be used to identify physiological features that co-vary with patient-reported PSM. These analyses are exploratory and hypothesis-generating: any patterns identified will be interpreted as correlates of patient-reported propagated sensation requiring independent replication, not as evidence of causality or as validation of PSM as a discrete biological entity.

## 2 Methods/design

### 2.1 Study design

This single-center, prospective observational cohort study will be conducted at Shenzhen Hospital (Futian) of Guangzhou University of Chinese Medicine. Participants diagnosed with lumbar disc herniation and radicular pain will be enrolled based on predefined criteria. Employing a naturalistic observation design, all participants will receive a standardized “lumbar-gluteal” acupuncture protocol. Without employing additional manipulation techniques beyond the standardized protocol, cohorts will be stratified based on the naturalistic occurrence of PSM during treatment into three groups: PSM-positive, PSM-negative, and indeterminate (see Section 2.4 for detailed definitions). The primary objectives will be to compare clinical outcomes and to explore the underlying neuroelectrophysiological and neuroinflammatory correlates. Detailed procedures and assessment time points are outlined in Figure 1.

**Figure 1:**
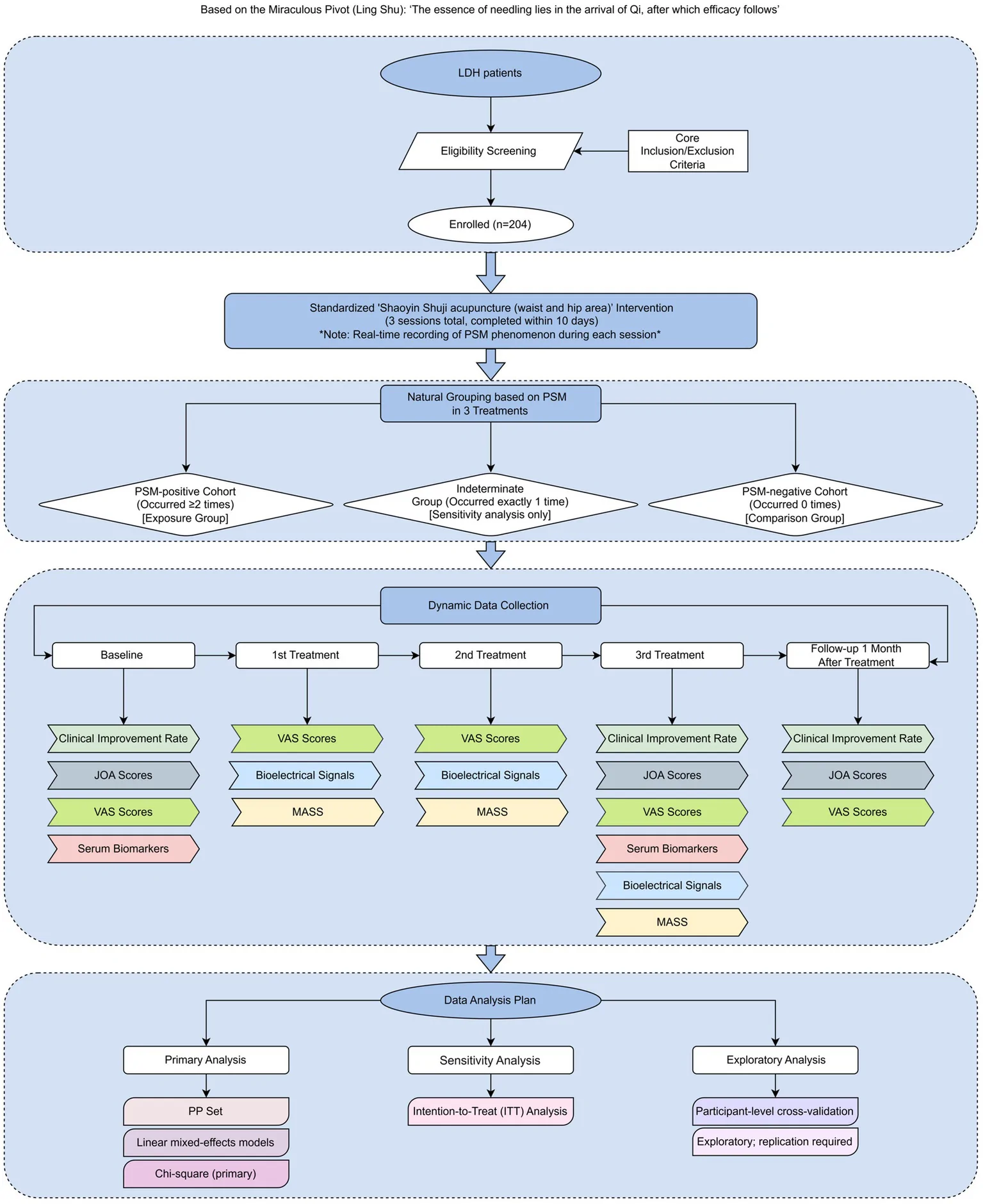
Study flow chart.

### 2.2 Study registration

This study was approved by the Ethics Review Committee of Shenzhen Hospital (Futian) of Guangzhou University of Chinese Medicine (No. GZYLL(KY)-2025-071) and has been prospectively registered with the International Traditional Medicine Clinical Trial Registry (ITMCTR) (ID: ITMCTR2025001534).

### 2.3 Patients

#### 2.3.1 Diagnostic criteria

The clinical diagnosis of lumbar disc herniation (LDH) with radiculopathy will be established in strict accordance with the North American Spine Society (NASS) Evidence-Based Clinical Guidelines. Eligible participants will be required to exhibit the following constellation of clinical and radiographic features:

1. persistent radicular pain radiating to the lower extremities;
2. a positive Straight Leg Raise (SLR) test (angle ≤ 60°);
3. motor deficits, defined as muscle strength ≤ Grade 4;
4. objective sensory abnormalities (paresthesia/hypoesthesia) or diminished deep tendon reflexes in the corresponding dermatome;
5. Magnetic Resonance Imaging (MRI) evidence of disc herniation morphologically consistent with the clinical presentation.

#### 2.3.2 Inclusion criteria

1. Meets the above diagnostic criteria for moderate lumbar disc herniation with nerve root involvement;
2. The herniated lumbar disc is located between L4 and S1;
3. Aged 18 to 65 years;
4. Voluntarily agrees to participate in this study and has signed the informed consent form.

#### 2.3.3 Exclusion criteria

1. Cognitive impairment or communication barriers precluding valid informed consent or accurate reporting of sensation (including PSM and pain intensity);
2. Absolute contraindications to acupuncture, such as severe coagulopathy or local skin infection at the treatment site;
3. Severe uncontrolled systemic comorbidities, including but not limited to advanced cardiovascular, hepatic, renal, or neurological dysfunction, as well as poorly controlled hypertension or diabetes;
4. History of spinal surgery within the preceding 3 months;
5. Radiographic evidence of sequestered or extruded disc herniation, or clinical signs indicative of Cauda Equina Syndrome;
6. Receipt of acupuncture, manual therapy, or analgesic medications that could confound pain assessment within 1 month prior to enrollment;
7. Current pregnancy or lactation;
8. Concurrent participation in another clinical trial or participation within 2 weeks prior to screening.

#### 2.3.4 Shedding criteria

1. Protocol Violation (Prohibited Interventions): Initiation of any prohibited concomitant treatments or medications not specified in the protocol that could confound the evaluation of therapeutic efficacy (including PSM assessment);
2. Intercurrent Illness: Emergence of serious adverse events or new medical conditions that render continuation of the study medically inadvisable or interfered with the treatment process;
3. Poor Compliance: Failure to complete the three scheduled treatment sessions without a documented valid medical justification;
4. Unauthorized Co-interventions: Receipt of concurrent physical therapies specifically targeting the lumbar region (e.g., Tuina, chiropractic manipulation, or physiotherapy) during the treatment phase.

#### 2.3.5 Stopping criteria

If severe adverse reactions or complications occur during treatment, it is considered unsuitable to continue observation.

#### 2.3.6 Recruitment method

1. Outpatient Recruitment (Primary Strategy):

Trained research coordinators will conduct preliminary screening of patients presenting with LDH at the Departments of Acupuncture and Pain Management within Shenzhen Hospital (Futian) of Guangzhou University of Chinese Medicine. Potential candidates meeting the initial eligibility criteria will be identified during their clinical visits and provided with a comprehensive introduction to the study.

1. Community Advertising (Supplementary Strategy):

To broaden the recruitment reach, recruitment posters will be strategically displayed on designated hospital bulletin boards and at affiliated Community Health Service Centers.

### 2.4 Cohort stratification

To maintain the integrity of the observational design, the acupuncturists will not employ specific manipulation techniques to actively induce the PSM phenomenon, nor will the assessors perform any additional provocation maneuvers (e.g., snapping palpation or exploratory needling) to elicit muscle twitches. Instead, participants will be stratified into cohorts after all three treatment sessions are completed (post-hoc stratification), based solely on the naturalistic manifestation of PSM during the treatment course. PSM will be assessed after each of the three treatment sessions using criteria that combine patient-reported measures with assessor-documented, sEMG-recordable needle-evoked lower-limb muscle twitches (see Section 2.5.4). In each session, the patient’s report of propagated sensation and the assessor’s documentation of involuntary lower-limb muscle twitches occurring in temporal association with needle manipulation are recorded independently, before classification. This dual-component approach will be adopted because the twitch response is observable by the assessor and time-locked to needle manipulation, thereby reducing the risk of misclassifying pre-existing or fluctuating radicular symptoms as PSM (19, 20). The classification criteria will be defined as follows:

1. *PSM-positive cohort*: Participants who report PSM in at least 2 out of 3 treatment sessions (≥2/3 sessions), with at least one of these reports accompanied by an assessor-documented, sEMG-recordable needle-evoked lower-limb muscle twitch.
2. *PSM-negative cohort*: Participants who report PSM in 0 out of 3 treatment sessions (0/3 sessions) and in whom no needle-evoked lower-limb muscle twitch is documented in any session.
3. *Indeterminate group*: Participants who report PSM in exactly 1 out of 3 treatment sessions, or whose subjective reports and twitch documentation are discordant (e.g., repeated PSM reports without any documented twitch, or documented twitches without any PSM report). This group will be excluded from the primary per-protocol analysis but may be included in exploratory sensitivity analyses and will be summarized in descriptive analyses to fully present the PSM occurrence patterns of all participants.

### 2.5 Interventions

This study will use a standardized Shaoyin Shuji acupuncture in the waist and hip area (28). Practitioners will perform routine Deqi manipulation (lifting-thrusting and twirling) as part of the standardized protocol; this is standard clinical practice rather than an attempt to “force” PSM. All acupuncture procedures will be performed by licensed practitioners with a minimum of 5 years of clinical experience, all of whom have undergone standardized training in the study protocol.

#### 2.5.1 Acupoints selection

Bilateral BL23 (Shenshu), BL24 (Qihaishu), BL25 (Dachangshu), GB30 (Huantiao), and two Ashi points selected at the most prominent tender points in the local lumbar region.

#### 2.5.2 Acupuncture needles

Disposable sterile acupuncture needles (0.40 mm × 75 mm, Huatuo brand, Medical Device Manufacturing License No. 20010020, Jiangsu Provincial Medical Products Administration) will be used in this study.

#### 2.5.3 Manipulation

Participants will be placed in a prone position, and the treatment sites will be sterilized with 75% alcohol. The intervention will employ the standardized Shaoyin Shuji acupuncture in the waist and hip area protocol. Needling specifications will be as follows:

1. BL23 (Shenshu), BL24 (Qihaishu), and BL25 (Dachangshu): Needles will be inserted obliquely at a 45-degree angle, directed medially toward the spine, to a depth of approximately 60 mm.
2. GB30 (Huantiao): Needles will be inserted perpendicularly to a depth of approximately 60 mm.
3. Ashi points: Needles will be inserted perpendicularly to a depth of 20–30 mm.

Following insertion, practitioners will perform manual manipulation to elicit Deqi as part of the standardized protocol, not to induce PSM artificially, but to observe the natural patient response to standardized needling. The manipulation protocol specifies the duration (30–60 s per point), rotating amplitude (90–180 degrees), lifting-thrusting amplitude (0.3–0.5 cm), and frequency (1–2 times per second) to ensure reproducibility across practitioners and sessions. The choice of reinforcing (Bu) or reducing (Xie) techniques will be guided by a pre-specified clinical reference framework based on traditional Chinese medicine pattern differentiation; this framework will be established and documented during practitioner training to ensure consistent decision-making across operators. Needles will be retained for 20 min.

#### 2.5.4 PSM assessment

PSM is defined as needling sensation (soreness, numbness, distension, or heaviness) radiating beyond the needling site along meridian pathways to the lower extremities (1, 2). PSM occurrence will be assessed using a standardized protocol combining a subjective component (patient-reported propagated sensation, temporally anchored to needle manipulation) with an objective component (assessor-documented, sEMG-recordable needle-evoked lower-limb muscle twitches).

1. *Subjective assessment.* Immediately after each needle manipulation session, a trained research assistant will administer a standardized structured interview: “Compared with your usual radicular symptoms, do you feel a new or different sensation (soreness, numbness, distension, or heaviness) propagating along your leg during the needle manipulation? If so, please describe the location, intensity, and duration of the propagation.” For any reported propagated sensation, its temporal characteristics will be recorded, including onset (time from needle insertion to first perception of the sensation), peak, and duration. PSM intensity will be rated by the participant on a 0–10 numerical rating scale (0 = no sensation, 10 = extremely strong propagation). When participants report no clear change from baseline, the sensation will be classified as indeterminate and excluded from the primary PSM grouping.
2. *Objective assessment.* Surface sEMG electrodes placed over the major lower-limb muscles of the affected limb, together with independent assessor documentation, will capture any involuntary muscle twitch occurring in temporal association with needle manipulation. No provocation maneuvers (e.g., snapping palpation or exploratory needling) will be performed to elicit twitches; only responses occurring in direct temporal association with standardized needle manipulation will be recorded (19, 20). Because twitch documentation is time-locked to needle manipulation, it provides an objectively observable anchor for distinguishing procedure-related PSM from pre-existing radicular symptoms. Sessions with discordant subjective and objective findings will be flagged accordingly.

#### 2.5.5 Treatment regimen

The treatment course will consist of three sessions completed within a 10-day window. The interval between consecutive sessions will be recommended to be 1–3 days, adjustable based on clinical feasibility.

#### 2.5.6 Practitioners

All procedures will be performed by licensed acupuncturists with a minimum of 5 years of clinical experience, strictly adhering to the study protocol.

#### 2.5.7 Standards and guidelines

This intervention is reported in accordance with the STRICTA 2010 guidelines. Acupoint locations will adhere to the WHO Standard Acupuncture Point Locations in the Western Pacific Region, and needling techniques will be based on the national planning textbook Science of Acupuncture and Moxibustion (China Press of Traditional Chinese Medicine).

### 2.6 Sample size calculation

The sample size will be determined based on the primary outcome measure—the clinical improvement rate derived from the Japanese Orthopaedic Association (JOA) scores. The primary analysis will compare the PSM-positive and PSM-negative cohorts; the indeterminate group will be excluded from this primary analysis and used only in exploratory sensitivity analyses. Data from our pilot study informed the improvement rates, which will be estimated at 85% for the PSM-positive cohort and 65% for the PSM-negative cohort.

Assuming a two-sided significance level of *α* = 0.05 and a statistical power of 1 − *β* = 0.90, the calculation for comparing two independent proportions indicated a minimum requirement of 90 participants per group. To account for a potential attrition rate of 15%, the final recruitment target will be adjusted to 102 participants per cohort, yielding a total sample size of 204.

### 2.7 Outcome measures

Clinical outcomes will be assessed using the Japanese Orthopaedic Association (JOA) and Visual Analogue Scale (VAS) scores, and propagated sensation will be quantified using the Massachusetts General Hospital Acupuncture Sensation Scale (MASS), which will be collected at each treatment session. The clinical improvement rate will be calculated based on the JOA scores for each participant. The schedule of all outcome assessments is summarized in Table 2.

**Table 2:** Schedule of outcome assessments.

Evaluation metrics	Screening	Baseline phase	Each treatment	Third treatment	1-month follow-up
Demographic data	×				
Inclusion/exclusion Criteria	×				
JOA scores		×		×	×
VAS scores		×	×	×	×
MASS			×		
Bioelectrical signal			×		
Serum collection		×		×	
Safety record			×	×	×

In parallel, multimodal bioelectrical signals will be acquired during each treatment session to enable real-time monitoring of physiological responses. Serum biomarkers will be measured before and after the intervention to capture molecular-level changes. These integrated assessments will be designed to enable multidimensional, dynamic monitoring of PSM.

#### 2.7.1 Clinical improvement rate

The primary outcome measure will be the clinical improvement rate, derived from the Japanese Orthopaedic Association (JOA) scores. JOA assessments will be conducted at three time points: baseline (immediately before the first treatment), post-intervention (immediately after the third treatment session), and at the one-month follow-up visit. The clinical improvement rate will be calculated using the following formula:

$$\text{Clinical Improvement rate}=\frac{\begin{array}{l} \text{Post}-\text{treatment scores}\\ -\mathrm{Pre}-\text{treatment scores} \end{array}}{29-\mathrm{Pre}-\text{treatment scores}}\times 100\%$$

Based on the improvement rate, treatment efficacy will be categorized according to the original Hirabayashi criteria (29) into four tiers: excellent (improvement rate ≥ 75%), good (50% ≤ improvement rate < 75%), fair (25% ≤ improvement rate < 50%), and poor (improvement rate < 25%).

For the primary analysis, differences in the primary outcome between the PSM-positive cohort and the PSM-negative cohort will be compared using the per-protocol set (excluding the indeterminate group). Secondary and exploratory analyses will employ tests for trend to examine potential dose–response relationships between the frequency of PSM occurrence and the degree of therapeutic efficacy. To assess the robustness of the primary findings, sensitivity analyses will be conducted in which the indeterminate group is alternately excluded, merged with the PSM-negative cohort, or merged with the PSM-positive cohort. In addition, a sensitivity analysis will be conducted following the intention-to-treat principle, including all enrolled participants.

#### 2.7.2 Secondary outcomes

1. Pain Intensity

Pain intensity will be quantified using the Visual Analogue Scale (VAS) (30). Participants will rate their pain on a continuum from 0 (“no pain”) to 10 (“worst pain imaginable”). Assessments will be conducted at baseline, immediately following each treatment session, and at the 1-month follow-up after the completion of the treatment course.

1. Lumbar Function

Functional status of the lumbar spine will be evaluated using the Japanese Orthopaedic Association (JOA) scores (total scores: 29) (31). Data will be collected at baseline, immediately upon completion of the third treatment session, and at the 1-month follow-up.

1. Standardized Acupuncture Sensation Assessment

The Massachusetts General Hospital Acupuncture Sensation Scale (MASS) will be administered to quantify the multi-dimensional characteristics of PSM. The MASS captures multiple dimensions of acupuncture-induced sensations, with particular emphasis on the Intensity subscale (rated 0–10) and the Spreading/Radiating subscale, which specifically assesses the extent to which the sensation travels beyond the needling site. A trained research assistant will administer the Chinese version of the MASS (C-MMASS) within 5 min after needle removal at each treatment session.

Patients will also be asked to indicate the location and extent of sensation propagation on a body diagram. The research assistant will measure the distance from the needling site (lumbar region) to the distal-most point of reported sensation using a standardized ruler. Distances will be categorized as: (a) lumbar region only; (b) to the buttock; (c) to the knee; (d) to the calf, or (e) to the foot.

Two trained research assistants will independently record PSM assessments, and inter-rater reliability (using Cohen’s Kappa coefficient) will be calculated to ensure data quality. A Kappa ≥ 0.80 is required before formal study enrollment begins.

MASS data will provide supplementary, descriptive characterization of PSM intensity and spreading.

##### 2.7.2.1 Multimodal biosignal features

1. Measurement equipment and parameter setting

To comprehensively capture the neurophysiological responses associated with the PSM phenomenon, two complementary bio-electrical acquisition systems will be employed.

First, peripheral physiological biomarkers will be monitored using the BiosignalsPlux multi-channel system (Plux Wireless Biosignals S.A., Portugal). To ensure objective and high-fidelity acquisition, sEMG sensors will be affixed to the lumbar region (Figure 2a), the lateral aspect of the calf (Figure 2b), and the posterior aspect of the calf (Figure 2c) of the affected limb to capture local muscle dynamics. The calf channels will provide the objective record of needle-evoked lower-limb muscle twitches used in PSM classification (see Sections 2.4 and 2.5.4), while the lumbar channels capture paraspinal muscle activity at the needled site. Dual-electrode EDA sensors will be positioned on the plantar surface to monitor skin conductance changes (Figure 2d), and horizontal/vertical EOG signals will be recorded via electrodes distributed peri-orbitally around the left eye (Figure 2e). The system will operate at a sampling rate of 1000 Hz utilizing disposable medical electrodes (Shenzhen Nanbei Pharmaceutical Co., Ltd.), with skin-electrode impedance strictly calibrated to <20 kΩ prior to data collection to maximize signal integrity.

**Figure 2:**
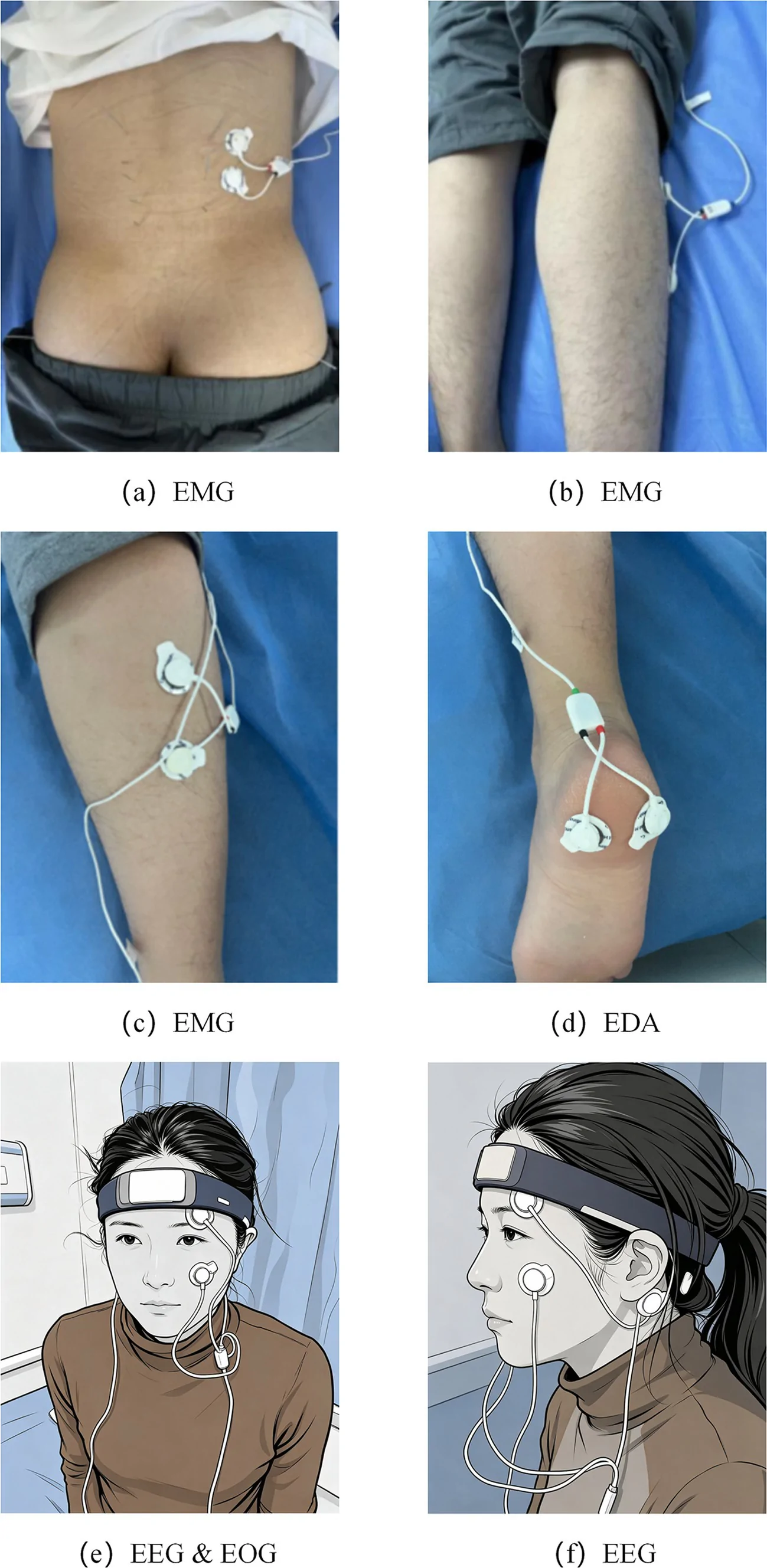
Representative placement of electrodes and sensors.

Simultaneously, central neural oscillations will be acquired using the Muse 2 wireless EEG headband (InteraXon Inc., Canada). This portable dry-electrode system will be configured to capture activity from the prefrontal and auricular regions, specifically targeting alpha (8–13 Hz), beta (13–30 Hz), and theta (4–8 Hz) frequency bands. The headband will be rigorously fitted to align with the frontal and mastoid regions; special care will be taken to center the sensor pod and minimize hair obstruction to facilitate optimal electrode-skin contact (Figure 2f). Following physical adjustment, a stabilization window of 30 to 60 s will be enforced to mitigate movement artifacts and calibrate the sensors prior to recording.

1. Measurement time

Multimodal bio-electrical signals will be recorded continuously throughout each acupuncture session using a standardized four-phase protocol. All recordings will be synchronized with procedural event markers, including needle insertion, the onset and offset of each manual manipulation episode, needle removal, and the onset of patient-reported PSM. Participants will remain in a prone position and be instructed to minimize voluntary movement and speaking during data acquisition.

1. Baseline Phase (5 min): Before needle insertion, participants will rest quietly in the prone position for 5 min. These recordings will provide a stable pre-intervention reference for autonomic, sensory, and motor activity.
2. Needle Manipulation Phase (1 min): Following needle insertion, standardized manual manipulation will be performed for 1 min according to a predefined procedure. The onset and offset of manipulation will be electronically marked in the recordings to characterize immediate physiological responses to needling. Participants will indicate whether PSM occurs using a response method that minimizes movement; the onset, distribution, direction, and intensity of PSM will be documented.
3. Needle Retention Phase (20 min): After manipulation, the needles will remain in place without further stimulation for 20 min. Continuous recording will be maintained to assess the persistence and temporal evolution of acupuncture-evoked physiological responses.
4. Post-needling Phase (5 min): Immediately after needle removal, recordings will continue for an additional 5 min while participants remain at rest. This phase will be used to evaluate post-stimulation effects and the return of physiological activity toward baseline.

##### 2.7.2.2 Serum biomarker analysis

To investigate the systemic immunomodulatory mechanisms associated with the PSM phenomenon, peripheral venous blood samples (5 mL) will be collected from participants at two distinct time points: at baseline (prior to the first intervention) and within 30 min following the completion of the third treatment session. Whole blood will be allowed to clot at room temperature prior to centrifugation at 1,000 × g for 10 min at 4 °C. The isolated serum will be immediately aliquoted into cryotubes and stored at −80 °C to ensure sample stability, with strict protocols in place to avoid repeated freeze–thaw cycles. A multiplex bead-based immunoassay will be subsequently utilized to quantify a panel of 12 cytokines, specifically IL-1*β*, IL-2, IL-4, IL-5, IL-6, IL-8, IL-10, IL-12p70, IL-17, IFN-γ, TNF-*α*, and IFN-*α*. Data from these biomarkers will be analyzed to explore longitudinal changes in immune profiles between the PSM-positive cohort and the PSM-negative cohort, and will be further integrated into the study’s multimodal analysis framework for exploratory assessment.

### 2.8 Adverse events monitoring and recording

All adverse events, including fainting, needle breakage, needle sticking, local hematoma, allergic reactions, infection, and other acupuncture-related discomforts, will be meticulously monitored and recorded. Appropriate management will be provided accordingly.

### 2.9 Data management and quality control

To ensure the authenticity, integrity, and confidentiality of the study data, strict management protocols will be implemented. Clinical assessment outcomes (including JOA and VAS scores) and laboratory results will be recorded in standardized paper Case Report Forms (CRFs). Data entry will adhere to standard operating procedures, requiring timely and accurate recording. Corrections will be made via a single strike-through accompanied by a date and signature/reason; the use of correction fluid will be strictly prohibited.

Digital multimodal biosignal data will be acquired directly through the BiosignalsPlux and Muse 2 systems, which automatically generate encrypted raw files to ensure data security. For manual data, a double-entry verification strategy will be employed: two trained research assistants will independently enter data from the paper CRFs into the electronic database. Any discrepancies identified during cross-validation will be resolved by referencing the original source documents. The database will distinguish participant-level, session-level, event-level, and signal-window-level records. Each participant, treatment session, needling event, and biosignal file will receive a non-identifying hierarchical code.

For this study, the exposure will be termed patient-reported propagated sensation (PSM), a neutral operational label rather than a validated biological state. Before each session, participants will mark the location, quality, and intensity of their ongoing radicular pain, numbness, heaviness, and paresthesia on a standardized body map. A session will be classified as PSM-positive only when the participant reports a new radiating or spreading sensation that (1) begins after needle insertion or manipulation, (2) extends continuously from a needled site to a furthest point at least 2 cm beyond that site, and (3) is distinguishable by the participant from the pre-session symptom map on the basis of onset, trajectory, location, or sensory quality. Reports that cannot be distinguished from pre-existing or fluctuating radicular symptoms will be coded as indeterminate and excluded from the primary PSM-positive versus PSM-negative comparison. A sensitivity analysis will apply a more conservative propagation threshold of 5 cm and will separately reclassify indeterminate sessions. Concordance with a predefined meridian map will be scored after data collection as a descriptive attribute and will not be required for PSM classification. Local Deqi sensations, practitioner-reported needle grasp, and traditional Qi-arrival judgments will be recorded separately and will not be used interchangeably with PSM.

All acquisition systems will be synchronized to a common study clock before each session. Electronic event markers will identify the start and end of baseline recording, needle insertion, each manipulation episode, PSM onset indicated by the participant and marked by a trained researcher, needle retention, needle removal, and post-needling recording. Clock offsets between devices will be measured at the beginning and end of each session and corrected during preprocessing. Practitioner identity, acupoint, insertion and manipulation timing, manipulation duration, and any protocol deviation will be recorded so that stimulation-related variation can be examined rather than attributed to PSM.

### 2.10 Statistical analysis plan

The analysis will be organized around two distinct questions: (1) whether physiological responses to standardized needling differ when a participant reports patient-reported propagated sensation (PSM), and (2) whether the frequency or intensity of PSM is associated with subsequent clinical outcomes. PSM is an operational patient report, not an independently validated biological state. EEG, sEMG, EDA, and EOG will therefore be treated as possible physiological correlates rather than as an objective reference standard. The core outcomes, preprocessing settings, exclusion rules, covariates, and multiplicity families will be finalized before database lock.

#### 2.10.1 Phase-based and clinical analysis

Analyses will be performed using SPSS 27.0 and R. Continuous variables will be summarized as mean and standard deviation when approximately normally distributed, or as median and interquartile range when skewed. Categorical variables will be reported as counts and percentages. Effect estimates will be presented with 95% confidence intervals, rather than relying only on *p* values. For the primary outcome (clinical improvement rate), comparisons between cohorts will be performed using the chi-square test, and effect estimates will be reported as risk differences with 95% confidence intervals.

Physiological changes will be examined across the four prespecified phases of each session: baseline, needle manipulation, needle retention, and post-needling. The main comparisons will be manipulation versus baseline (immediate response), retention versus baseline (sustained response), post-needling versus baseline (after-effect), and post-needling versus manipulation (recovery toward baseline). Signal features will first be expressed as change from the same session’s baseline so that each participant serves as his or her own reference.

Because participants contribute repeated phases and up to three sessions, linear mixed-effects models will be used. These models allow physiological changes between phases to be compared while accounting for the correlation among repeated measurements from the same participant and for individual differences in baseline physiological activity. Skewed outcomes will be transformed or examined with robust or rank-based sensitivity analyses.

PSM will initially be analyzed as a session-level, time-varying exposure. Each session will be classified according to the PSM report obtained during that session, without using information from subsequent sessions. For clinical outcomes assessed after multiple sessions, PSM exposure will be summarized using the cumulative number and proportion of PSM-positive sessions and the mean reported intensity up to each assessment. Changes in VAS and JOA scores will be examined using repeated-measures models to explore their associations with the occurrence, frequency, and intensity of PSM.

Missing physiological windows will not be imputed. The amount and reasons for missing or rejected data will be reported by signal modality, recording phase, and PSM report category. False discovery rate correction will be applied separately within each signal family and analysis question. Results will be reported primarily as effect estimates with 95% confidence intervals. Analyses of cytokines, individual sensory descriptors, and clinical outcome prediction will be considered exploratory.

#### 2.10.2 Patient-reported PSM and event-related analysis

During recording, participants will indicate the first onset of a suspected propagated sensation using a standardized minimal-movement reporting procedure. Immediately after this indication, a trained researcher, rather than the participant, will trigger a synchronized PSM event marker using a dedicated event-marker button. This marker will serve only as a temporal label for PSM-specific detection and analysis and will not be considered independent confirmation of PSM. Practitioner-reported needle grasp or Qi arrival will be recorded separately and will not replace the participant’s self-report.

Before each session, participants will mark the location, quality, and intensity of their usual radicular pain, numbness, heaviness, and paresthesia on a standardized body map, as specified in Section 2.9. After needling, participants will confirm whether the reported sensation was new or clearly increased, where it began, how far it spread, its intensity and trajectory, and whether it differed from the baseline symptom map. A PSM label will be assigned only when the report meets the prespecified spatial and temporal criteria in Section 2.9. Reports that cannot be reliably distinguished from pre-existing or fluctuating radicular symptoms will be classified as indeterminate.

For PSM reports with a reliable onset time, event-related analyses will compare the intervals immediately before and after the synchronized PSM marker. Because the signal modalities differ in temporal resolution and response latency, the prespecified interval length will differ by modality: 2 s for EEG, 5 s for sEMG (see Section 2.11.2), and 10 s for EDA (see Section 2.11.3). In each modality, the post-marker interval will first be compared with the pre-marker interval to characterize immediate physiological changes associated with the reported onset of PSM. These two intervals will also be compared with reference values calculated from artifact-free 5-min windows during the pre-needling baseline phase and the post-needling resting phase. PSM status will not be assigned in advance, and no pseudo-events will be created for sessions without PSM. A PSM event marker will be added only when propagated sensation is actually reported and subsequently confirmed as meeting the prespecified criteria. Sessions without PSM will contribute to the phase-based baseline, stimulation, retention, and post-needling comparisons but not to the PSM event-locked analysis. Reports with uncertain onset timing will also be excluded from the event-locked analysis. Any speech or movement occurring around the marker will be documented, and contaminated EEG or sEMG intervals will be excluded from the corresponding analysis.

### 2.11 Signal-specific biosignal processing and multimodal analysis

Each modality will be processed with a signal-specific pipeline because EEG, sEMG, EDA, and EOG have different units, frequency ranges, artifacts, and physiological meanings. The original recordings will remain unchanged. Processing code, software versions, filter settings, rejected segments, and usable recording time will be logged to support reproducibility. Device clocks and procedural markers will be aligned before segmentation.

Given that EEG, sEMG, EDA, and EOG differ fundamentally in frequency content, units, artifact structure, and physiological interpretation, separate preprocessing pipelines will be applied to each signal type. The preprocessing parameters for each signal modality are summarized in Table 3, and the overall workflow of feature extraction and multimodal integration is presented in Figure 3.

**Table 3:** Summary of signal-specific preprocessing and feature extraction.

Signal	Filtering	Artifact removal	Segmentation	Additional processing
EEG	0.5–45 Hz	Flat-channel detection; EOG-assisted correction; rejection of epochs exceeding ±100 μV	5-s epochs, 50% overlap	Morlet wavelets
EOG	0.1–15 Hz	Blink and movement detection; drift and saturation rejection	5-s epochs, 50% overlap	Used primarily for EEG artifact monitoring
sEMG	20–450 Hz	Saturation, electrode-loss, needle-handling, and movement artifact rejection	1-s epochs, 50% overlap	Full-wave rectification + RMS envelope (100 ms window)
EDA	0.05–5 Hz	Motion-spike, saturation, and electrode-loss detection	10-s epochs, 50% overlap	cvxEDA; SCL and SCR features

**Figure 3:**
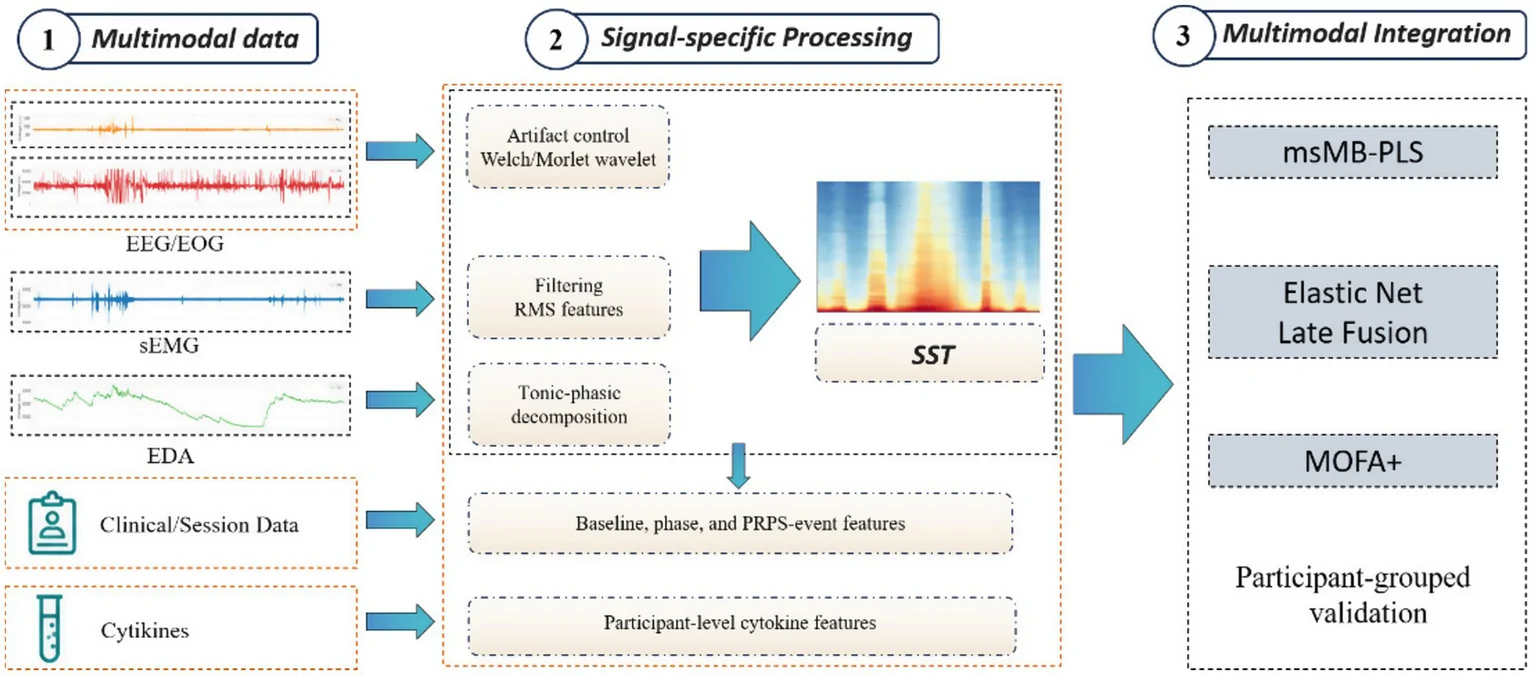
Multimodal analysis workflow for patient-reported propagated sensation.

#### 2.11.1 EEG and EOG preprocessing and features

EEG will be analyzed at the Muse 2 native sampling rate (256 Hz). The device-native reference will be retained and documented. Signals will be de-meaned and zero-phase band-pass filtered from 0.5 to 45 Hz. Flat channels, contact loss, saturation, large amplitude transients, and excessive high-frequency activity will be identified with robust channel- and epoch-level thresholds.

Horizontal and vertical EOG will be de-trended and band-pass filtered from 0.1 to 15 Hz. Blinks, saccades, slow drift, saturation, and large facial or head movements will be detected using robust amplitude and slope thresholds, followed by review of uncertain events. EOG will be used primarily to identify ocular contamination in EEG. Blink rate, blink amplitude, and EOG root-mean-square activity may be reported as secondary indicators of ocular activity, arousal, or movement.

Because the headset provides only a small number of EEG channels, independent component analysis will not be used as the default ocular-artifact method. Instead, EOG-assisted artifact marking or regression and rejection of contaminated epochs will follow principles developed for low-density EEG processing.

For phase comparisons, artifact-free epochs will be used to calculate log-transformed absolute and relative theta (4–8 Hz), alpha (8–13 Hz), and beta (13–30 Hz) power with Welch’s method. Event-related spectral changes will be estimated with complex Morlet wavelets, with temporal and frequency smoothing reported explicitly. Wavelet-based synchrosqueezing transform (SST) will be retained as a secondary analysis of artifact-clean EEG event windows to sharpen time-frequency ridges and estimate transient peak frequency and ridge energy.

#### 2.11.2 sEMG preprocessing and features

Raw sEMG will be de-meaned and zero-phase band-pass filtered from 20 to 450 Hz. Saturation, electrode detachment, abrupt baseline shifts, and transients caused by needle handling, button pressing, or whole-body movement will be flagged. Accepted signals will be full-wave rectified, and a 100-ms root-mean-square envelope will be calculated.

Prespecified sEMG features will include root-mean-square amplitude, integrated sEMG, median frequency, activation onset, burst duration, and change from the same session’s baseline for the lumbar and affected-leg channels. For reliably marked PSM events, features from the 5-s pre-marker and 5-s post-marker intervals will be compared with each other and with the pre-needling baseline and post-needling resting values. Wavelet-based SST may be applied secondarily to clean event windows to describe transient sEMG bursts.

#### 2.11.3 EDA preprocessing and features

EDA recordings will be converted to skin conductance in microsiemens and visually examined for saturation, electrode detachment, abrupt signal shifts, and motion spikes. After low-pass filtering at 5 Hz, the signals will be downsampled to 10 Hz using appropriate anti-alias filtering.

Skin conductance will be separated into tonic and phasic components using cvxEDA. Prespecified tonic features will include mean skin conductance level and its temporal slope. Phasic features will include the number, amplitude, area under the curve, and latency of skin conductance responses. Because electrodermal responses may develop more slowly than EEG or sEMG changes, EDA will be analyzed using an extended 10-s post-marker window.

For PSM events with a reliable onset marker, EDA features will be calculated separately for the 10-s intervals immediately before and after the marker. The post-marker values will be compared with the pre-marker values and with reference values obtained during the pre-needling baseline and post-needling resting phases.

#### 2.11.4 Synchrosqueezing transform

EEG and sEMG signals surrounding patient-reported PSM events may contain transient and non-stationary changes. Conventional time-frequency methods may produce spectral smearing. Therefore, complex Morlet wavelet analysis will be used as the primary time-frequency method, while wavelet-based synchrosqueezing transform (SST) will be applied as a secondary analysis to obtain a sharper time-frequency representation.

To capture the non-stationary characteristics of bioelectrical signals, we will first apply the STFT to convert the 1D time-series signal into a time-frequency representation. The STFT of a signal x(t) is defined as:

$$G_{x}(t,\omega )=\int_{-\infty }^{\infty }x(\tau )g(\tau -t)e^{-\mathrm{j\omega \tau}}\mathrm{d\tau}$$

Where $G_{x}(t,\omega )$ denotes the resulting complex-valued time-frequency representation of signal; x(τ) represents the original input bioelectrical signal (e.g., sEMG or EEG); g(τ–t) is the window function (such as a Gaussian window) centered at time t ; τ is the integration variable in the time domain; t denotes the time-shift parameter corresponding to the center of the sliding window; ω represents the angular frequency; and j is the imaginary unit defined as $\sqrt{-1}$ .

While STFT provides time-frequency localization, it is subject to the Heisenberg uncertainty principle (smearing). To improve resolution, we will utilize the Synchrosqueezed Transform (SST). SST reassigns the energy from the STFT to the instantaneous frequency.

The instantaneous frequency $\hat{\omega }(t,\omega )$ is estimated as:

$$\;\hat{\omega }(t,\omega )=-j\frac{\partial_{t}G_{x}(t,\omega )}{G_{x}(t,\omega )}$$

Where $\partial_{t}G_{x}(t,\omega )\;$ is the partial derivative of the STFT with respect to time t.

The final SST, denoted as $T_{s}(t,\xi )$ , is then computed by integrating the STFT energy over frequency lines that converge to the same instantaneous frequency:

$$T_{s}(t,\xi )=\int_{|\omega -\xi |<\delta }G_{x}(t,\omega )\mathrm{d\omega}$$

Where $T_{s}(t,\xi )$ denotes the synchrosqueezed time–frequency representation; ξ represents the reassigned frequency variable after energy reallocation; δ is a small threshold parameter that determines the width of the frequency bin used during reassignment; and the condition $|\hat{\omega }(t,\omega )-\xi |<\delta$ specifies that only those STFT coefficients whose estimated instantaneous frequency $\hat{\omega }(t,\omega )$ falls within a δ -neighborhood of ξ are grouped and reassigned to that frequency bin.

SST will be applied separately to artifact-free EEG and sEMG recordings from reliably marked PSM events. For each event, transient peak frequency, ridge energy, and ridge duration will be extracted from the 5-s pre-marker and 5-s post-marker intervals, which are wider than the primary event-locked windows for EEG (2 s; Section 2.10.2) because ridge tracking requires longer continuous segments. These features will be compared between the two event intervals and with the pre-needling baseline and post-needling resting values.

#### 2.11.5 Multilevel sparse multi-block partial least squares

Multilevel sparse multi-block partial least squares (msMB-PLS) will be used as the principal exploratory integration method for correlated features measured repeatedly across modalities. Prespecified blocks will comprise EEG/EOG features, lumbar and affected-leg sEMG features, EDA features, and clinical/session variables. Cytokine changes will not be copied across sessions; because they are measured only before treatment and after the third session, they will enter a separate participant-level supplementary analysis.

For participant i , session j , and modality block m , the feature vector will be decomposed into between-participant and within-participant components:

$$x_{\mathrm{ij}}^{\left(m\right)}={\bar{x}}_{i}^{\left(m\right)}+x_{\mathrm{ij},W}^{\left(m\right)}$$

Where

$${\bar{x}}_{i}^{\left(m\right)}=\frac{1}{n_{i}}\sum_{j=1}^{n_{i}}x_{\mathrm{ij}}^{\left(m\right)},\;\;\;\;x_{\mathrm{ij},W}^{\left(m\right)}=x_{\mathrm{ij}}^{\left(m\right)}-{\bar{x}}_{i}^{\left(m\right)}$$

here, $n_{i}$ is the number of available sessions for participant i , ${\bar{x}}_{i}^{\left(m\right)}$ represents the participant-specific mean for modality m , and $x_{\mathrm{ij},W}^{\left(m\right)}$ represents the session-specific deviation from that mean. The within-participant components will be stacked to form the matrix $X_{W}^{\left(m\right)}$ . This decomposition separates stable between-participant differences from session-to-session changes and follows the multilevel approach for paired or repeated-measures data.

Time-invariant variables, such as age, sex, and baseline disease characteristics, will remain in a separate between-participant clinical block. Prespecified transformations will be applied to strongly skewed features. Within each training set, features will be centered and scaled, and each block will be normalized to prevent blocks with more variables or larger variances from dominating the analysis solely because of their scale.

For each modality block, a latent score will be constructed as

$$t_{m}=X_{W}^{\left(m\right)}w_{m},$$

Where $w_{m}$ is the sparse loading vector for block m . Let u denote the response score. For a single outcome, u=y , where y represents either binary PSM occurrence, continuous PSM intensity, or a standardized clinical change score. The simplified msMB-PLS objective will be

$$\underset{\left\{w_{m}\right\}}{\mathrm{maximize}}[\sum_{m<n}c_{\mathrm{mn}}{\mathrm{cov}}^{2}(t_{m},t_{n})+\sum_{m}c_{\mathrm{mY}}{\mathrm{cov}}^{2}(t_{m},u)],$$

subject to

$${\left\|\;w_{m}\right\|}_{2}=1,\;{\left\|w_{m}\right\|}_{1}\leq s_{m}.$$

The design coefficient $c_{\mathrm{mn}}$ determines whether modality blocks m and n are connected, while $c_{\mathrm{mY}}$ determines whether block m is connected to the response. The $L_{2}$ constraint normalizes each loading vector, whereas the $L_{1}$ constraint controlled by $s_{m}$ produces sparse loadings and retains a smaller, more interpretable set of features. Squared covariance is used so that components with opposite loading directions do not cancel one another.

Overall, msMB-PLS will characterize coordinated cross-modal variation and identify a concise set of features contributing to the shared latent components. However, because msMB-PLS estimates the modality blocks jointly, it may be difficult to determine whether predictive information arises from one modality or from the combination of several modalities. Therefore, a complementary modality-specific strategy will next be used to quantify the independent contribution of each data source and determine whether combining their predictions improves performance.

#### 2.11.6 Modality-specific elastic-net models and late fusion

Modality-specific elastic-net models followed by late fusion will be used as a complementary supervised integration strategy. Separate elastic-net models will be fitted for the EEG/EOG, sEMG, EDA, and clinical/session blocks. Elastic net is suitable for these data because it combines variable selection with stabilization of correlated predictors, reducing overfitting while retaining groups of related physiological features. Modeling each modality separately also prevents a large feature block from dominating the analysis and allows its individual contribution to be evaluated.

Analyzing each modality separately also prevents a modality containing more features or larger numerical values from dominating the model. It enables direct comparison of the information contributed by central electrophysiological activity, muscular responses, autonomic activity, and clinical characteristics. Their predictions will subsequently be combined through late fusion, allowing complementary information to be integrated without merging all original features into a single high-dimensional matrix.

For modality m , let $x_{i}^{\left(m\right)}$ denote the feature vector for observation i , and let $y_{i}$ denote the corresponding PSM report or clinical outcome. The elastic-net coefficients will be estimated by minimizing the penalized loss:

$$({\overset{ˆ}{\beta }}_{0m},{\overset{ˆ}{\beta }}_{m})=\underset{\beta_{0m},\beta_{m}}{\arg\;\min}\{\begin{array}{l} -\frac{1}{N}\sum_{i=1}^{N}\ell [y_{i},\beta_{0m}+x_{i}^{(m)T}\beta_{m}]\\ +\lambda_{m}[\alpha_{m}{\left\|\beta_{m}\right\|}_{1}+\frac{1-\alpha_{m}}{2}{\left\|\beta_{m}\right\|}_{2}^{2}] \end{array}\}.$$

Where, ℓ(·) is the log-likelihood contribution, N is the number of observations, and $\lambda_{m}$ controls the overall strength of regularization. The mixing parameter $\alpha_{m}$ controls the balance between the two penalties. The $L_{1}$ component performs variable selection, whereas the $L_{2}$ component stabilizes coefficients when predictors are correlated. Thus, elastic net can retain a small but coherent group of related physiological features instead of selecting only one arbitrary feature from a correlated set.

For session-level PSM analysis, only phase-level features available in PSM-positive sessions will be included. PSM-centered event features will be analyzed separately because they are defined only after a PSM event has been reported.

Each modality-specific model will generate out-of-fold predictions. Cross-validation will be stratified at the participant level, so that all sessions from one participant remain within the same fold. Hyperparameters will be selected by inner cross-validation within the training folds only, and all preprocessing will likewise be performed within the training folds. Given the limited sample size relative to the number of features, no separate test set will be reserved; performance will be assessed solely from out-of-fold predictions, and features retained by these models will be reported as exploratory, pending replication in independent cohorts. For binary PSM analysis, these probabilities will be combined using a regularized logistic meta-model:

$${\overset{ˆ}{p}}_{i}^{\text{fusion}}={\text{logit}}^{-1}[\gamma_{0}+\sum_{m=1}^{M}\gamma_{m}\text{logit}({\tilde{p}}_{i}^{\left(m\right)})],$$

Where M is the number of modality blocks, $\gamma_{m}$ is the fusion weight assigned to modality m , and

$${\tilde{p}}_{i}^{\left(m\right)}=\min[1-\varepsilon ,\max(\varepsilon ,{\overset{ˆ}{p}}_{i}^{\left(m\right)})]$$

is a truncated probability used to avoid infinite logit values when a predicted probability is close to zero or one. For continuous VAS or JOA outcomes, the corresponding modality-specific predictions will be combined using a regularized linear meta-model.

PSM models will be evaluated mainly using balanced accuracy, ROC area, precision–recall area, Brier score, and calibration. Clinical-outcome models will report mean absolute error, root-mean-square error, and $R^{2}$ . The results will show whether different modalities provide complementary predictive information.

Because msMB-PLS and elastic-net fusion both use prespecified response variables, a complementary unsupervised analysis is required to examine multimodal structure without PSM or clinical-outcome labels.

#### 2.11.7 MOFA+ latent factor analysis

MOFA+ will therefore be used as a complementary unsupervised method to identify shared and modality-specific sources of variation without incorporating PSM labels or clinical-response outcomes into latent-factor estimation. This label-free approach reduces circularity and allows multimodal physiological structure to be identified before its relationship with patient-reported PSM is examined.

For modality m, the MOFA+ model is

$$X^{\left(m\right)}=Z{\left[W^{\left(m\right)}\right]}^{T}+E^{\left(m\right)},$$

Where $X^{\left(m\right)}$ is the standardized feature matrix, Z contains participant-level factor scores, $W^{\left(m\right)}$ contains modality-specific feature loadings, and $E^{\left(m\right)}$ represents residual variation. Sparsity and relevance priors allow each factor to capture variation shared across several modalities or variation primarily associated with one modality.

The model will use one row per participant. EEG/EOG, sEMG, and EDA features will be summarized using prespecified phase contrasts and across-session means or variability. After the unsupervised model has been finalized, factor scores will be tested separately for associations with cumulative PSM occurrence or intensity and with changes in VAS or JOA scores. These analyses will use covariate-adjusted regression with false discovery rate correction.

Agreement among MOFA+ factors, msMB-PLS components, and modality-specific findings will be described as convergence across analyses rather than independent confirmation, because all results are derived from the same cohort. Together, these methods provide complementary views of cross-modal covariance, predictive information, and label-free latent structure while maintaining an exploratory interpretation of the physiological correlates of patient-reported PSM.

## 3 Discussion

This study proposes a prospective cohort design integrated with multimodal systems-level physiological monitoring to ask three progressive questions: whether patient-reported PSM is associated with differential clinical outcomes, what physiological features characterize the PSM state, and whether these features can be identified reliably from multimodal measures.

Acupuncture is widely used for LDH. However, the heterogeneity of therapeutic responses remains a critical bottleneck restricting its standardization and mechanistic characterization. Emerging evidence suggests that PSM may be associated with enhanced acupuncture efficacy. Notably, therapeutic protocols explicitly incorporating maneuvers to facilitate needle sensation transmission have reported higher effective rates (89.1%) compared to routine acupuncture (71.1%) (32), and have achieved aggregate efficacy rates of 92.31% when combined with pharmacotherapy (33). However, in these precedents, PSM functioned merely as a component of a composite intervention rather than a controlled independent variable. Consequently, the observed variance in outcomes reflects the holistic effect of the combined therapies, precluding the precise quantification of the independent contribution of PSM. This methodological limitation, compounded by the prevalence of small-sample, single-center designs and the lack of objective tools to define and evaluate the PSM state, has prevented the rigorous validation of its clinical significance, thereby perpetuating the hiatus between traditional empirical wisdom and modern evidence-based medicine.

To overcome the methodological limitations of previous studies, this research will implement a major design adjustment by adopting a prospective cohort study framework. Instead of attempting to artificially randomize participants based on the inherently difficult-to-operationalize variable of PSM, we will treat the naturally occurring PSM phenomenon during standardized acupuncture intervention as the core exposure factor. This “naturalistic observational design” allows for a realistic assessment of the association between PSM status and clinical outcomes, thereby enabling an exploration of whether PSM is associated with differential clinical outcomes in a naturalistic setting.

Crucially, recognizing that associations do not establish causality, we will further integrate a multimodal analytical strategy. We will synchronously acquire multidimensional data, including neurophysiological signals (EEG, sEMG), autonomic responses (EDA), and systemic inflammatory cytokines, to explore physiological features that may be associated with patient-reported PSM. This approach will be intended to identify potential physiological patterns by exploring integrated neurophysiological and immune response patterns, without presuming a specific mechanistic hypothesis for the ‘efficacy follows Qi arrival’ principle.

We will compare the PSM-positive and PSM-negative cohorts to determine whether reductions in pain intensity (VAS scores) and improvements in lumbar function (JOA scores) at post-treatment and follow-up differ between the two groups. Concurrently, we will examine whether patterns of alpha (*α*), theta (θ), and beta (*β*) wave activities in the prefrontal cortex, as well as changes in cytokine profiles (pro-inflammatory and anti-inflammatory markers), differ between PSM-positive and PSM-negative participants.

The principal innovation of this study lies in extending the observational dimension of PSM from traditional static, discrete observations to dynamic, multimodal monitoring that encompasses the entire acupuncture treatment cycle: including baseline, needle manipulation, needle retention, and post-needling phases. A further innovation concerns the operationalization of PSM itself: because radicular symptoms inherent to LDH can be confused with propagated sensation, reports are anchored to the needle-manipulation phase and interpreted against baseline symptoms, ambiguous reports are classified as indeterminate, and needle-evoked lower-limb twitches documented by the assessor on calf sEMG channels provide an objective anchor independent of the patient’s sensory vocabulary (19, 20). This dual-component strategy reduces, but does not eliminate, misclassification.

Machine learning algorithms will be employed to explore patterns in the multidimensional dataset, with the objective of identifying physiological features associated with patient-reported PSM. These analyses are exploratory and hypothesis-generating. Because the reference standard is the patient’s report rather than a validated biological marker, any patterns identified are correlates of patient-reported PSM and do not independently establish PSM as a discrete neurobiological entity. As a supplementary analysis, unsupervised latent-factor analysis performed without reference to PSM labels (Section 2.11.7) will test whether the natural structure of the multimodal data corresponds to the subjective classification; concordance would provide convergent evidence, whereas discordance would itself be informative. These analyses may inform the future development of auxiliary assessment tools.

The cohort database, which includes clinical, electrophysiological, and biochemical data, can support future studies on predictors of therapeutic response and patient subtyping to guide personalized acupuncture protocols.

Alternative interpretations of the physiological findings also warrant consideration. The EEG, sEMG, EDA, EOG, and cytokine measures may capture responses unrelated to PSM, such as reactions to needling, pain, attention, expectation, or muscle activity. The four-phase recording protocol partly addresses this: comparing each participant with their own baseline helps separate responses linked to needle manipulation from general effects of needling. Prior work has also documented measurable physiological correlates of PSM using somatosensory-evoked potential mapping, infrared thermography, and gene expression profiling (11, 16, 17), which supports the plausibility of identifying such patterns in the present data. Even so, alternative explanations of any group differences cannot be excluded.

This study has several limitations. First, although prospective in design, the study is observational and cannot establish causality. The association between PSM and clinical outcomes may be confounded by unmeasured variables. Causal inference would require randomized controlled trials with PSM as a stratification factor. Second, as discussed above, the physiological measures may capture responses that are not specific to PSM, and alternative explanations of any group differences cannot be excluded. Third, PSM classification relies partly on subjective reports; real-time recording during needle manipulation and assessor-documented, sEMG-recordable muscle twitches reduce this dependence, but the MASS remains a post-hoc recall measure subject to recall bias. Accordingly, the term PSM is used throughout this protocol as a descriptive operational definition of what patients report, without presuming a specific biological mechanism or clinical meaning. Fourth, as a single-center study conducted at Shenzhen Hospital (Futian) of Guangzhou University of Chinese Medicine, the generalizability of findings remains uncertain; multi-center, large-scale studies are needed to replicate these findings, and future neuroimaging or metabolomics work may further characterize the underlying mechanisms. Fifth, the sample size was calculated for the primary clinical endpoint and is small relative to the number of features in the multimodal dataset. The machine-learning analyses are therefore preliminary and exploratory. Participant-level cross-validation, with hyperparameters selected within the training folds (Section 2.11.6), limits data leakage and pseudoreplication but cannot rule out overfitting; any features these models identify will require replication in independent cohorts.

This preliminary exploratory study examines the clinical and physiological correlates of patient-reported PSM in lumbar disc herniation (LDH) by integrating multimodal neurophysiological monitoring with molecular measurements. At most, it may identify physiological correlates of patient-reported PSM; it cannot provide independent neurobiological validation, nor establish PSM or any measured feature as an objective biomarker. All findings should be regarded as preliminary and hypothesis-generating, requiring independent replication. The findings may inform the design of future larger-scale, confirmatory studies of acupuncture efficacy for LDH and provide a methodological basis for quantifying propagated needling sensations.

## References

[1] Xu J Zheng S Pan X Zhu X Hu X. The existence of propagated sensation along the meridian proved by neuroelectrophysiology. Neural Regen Res. (2013) 8:2633–40. doi: 10.3969/j.issn.1673-5374.2013.28.005, 25206574 PMC4146028

[2] Zhang W-B Zhao Y Fuxe K. Understanding propagated sensation along meridians by volume transmission in peripheral tissue. Chin J Integr Med. (2013) 19:330–9. doi: 10.1007/s11655-013-1456-0, 23674110

[3] Song TT Wang J. Talking about the key factors to improve the therapeutic effect of acupuncture from “the arrival of qi ensures curative effect” (in Chinese). Chinese Acupuncture & Moxibustion. (2019) 39:197–200. doi: 10.13703/j.0255-2930.2019.02.023, 30942041

[4] Yin CS Chae Y Kang OS Kim S-T Hahm D-H Park J-Y . Deqi is double-faced: the acupuncture practitioner’s and the subject’s perspective. Evid Based Complement Alternat Med. (2015) 2015:1–6. doi: 10.1155/2015/635089, 26689128 PMC4672129

[5] Yuan H-W Ma L-X Zhang P Lin C Qi D-D Li J . An exploratory survey of Deqi sensation from the views and experiences of Chinese patients and acupuncturists. Evid Based Complement Alternat Med. (2013) 2013:430851. doi: 10.1155/2013/430851, 24348700 PMC3857737

[6] Xu Q Tang PP Wang XJ Mao JF Xu AJ Li HM . Origin and development of Hua Tuo's acupuncturing sensation technique (in Chinese). Chin Acupunct Moxibustion. (2021) 41:570–4. 10.13703/j.0255-2930.20200328-k0003 34002576

[7] Kong J Gollub R Huang T Polich G Napadow V Hui K . Acupuncture de qi, from qualitative history to quantitative measurement. J Altern Complement Med. (2007) 13:1059–70. doi: 10.1089/acm.2007.0524, 18166116

[8] Xu J Tang G Zhu X Lu Z Dong Y Gao H . Applying artificial intelligence technology to analyze objective manifestations of propagated sensation along meridians (in Chinese). Chin Arch Tradit Chin Med. (2026) 44:1–7. doi: 10.13193/j.issn.1673-7717.2026.06.001

[9] Hu X Wang P Xu J Wu B Xu X. The main characteristics of infrared radiant track along meridian courses over human body surface and the condition of its appearance. J Infrared Millim Waves. (2001) 20:325–328+402-404.

[10] Chen M Wu Z Hu X Xu J. Effect of acupuncture on partial oxygen pressure in different depths of tissues along governor vessel in 61 normal volunteer subjects. China J Tradit Chin Med Pharm. (2011) 26:1856–8.

[11] Hu X Wang P Xu J Xu X Wu B Gao L. Investigation on the thermal-source of infrared radiant track along meridian course over the human body surface and the mechanism of its formation. Acupunct Res. (2002) 27:258–9.

[12] Hu X Xu J Wang P Xu X Gao L Wu B. Preliminary exploration on the mechanism underlying the formation of infrared radiant track along meridian courses over the human body surface. J Infrared Millim Waves. (2003) 22:175–180+242-243.

[13] Xu J Pan X Hu X Wu B. Comparison between governor Meridian and its bilateral control points in microcirculatory blood perfusion in 53 volunteer subjects (in Chinese). Zhen Ci Yan Jiu. (2008) 33:321–5. 19097504

[14] Xu J Hu X Wang P Ye L Yang J. Comparison of the thermal conductivity of the related tissues along the meridian and the non-meridian (in Chinese). Zhongguo Zhen Jiu. (2005) 25:477–82. 16309135

[15] Xu J Hu X Wang P Wu B. Effect of acupuncture on infrared radiant track along meridians over the human body surface (in Chinese). Zhen Ci Yan Jiu. (2002) 27:255–8.

[16] Xu J Pan X Zheng S Hu X Sa Z. Comparison of topographical mapping of cortical somatosensory evoked response during propagated sensation along Meridian and imitating propagated sensation along Meridian (in Chinese). Fujian Zhong Yi Xue Yuan Xue Bao. (2010) 20:4–6+73.

[17] Zhu X Xu J Wang J Sa Z Pan X Zheng S . Analysis of the mechanism of propagated sensation along meridians based on gene expression profiles. J Tradit Complement Med. (2023) 13:236–44. doi: 10.1016/j.jtcme.2023.01.004, 37128199 PMC10148120

[18] Kou W Gareus I Bell JD Goebel MU Spahn G Pacheco-López G . Quantification of DeQi sensation by visual analog scales in healthy humans after immunostimulating acupuncture treatment. Am J Chin Med. (2007) 35:753–65. doi: 10.1142/S0192415X07005247, 17963316

[19] Jin G. Targets,Mechanisms,Techniques and clinical significance of twitch-eliciting acupuncture (in Chinese). Chin Arch Tradit Chin Med. (2026) 44:13–22. doi: 10.13193/j.issn.1673-7717.2026.05.003

[20] Rha D-W Shin JC Kim Y-K Jung JH Kim YU Lee SC. Detecting local twitch responses of myofascial trigger points in the lower-back muscles using ultrasonography. Arch Phys Med Rehabil. (2011) 92:1576–1580.e1. doi: 10.1016/j.apmr.2011.05.005, 21839982

[21] Koes BW Van Tulder MW Peul WC. Diagnosis and treatment of sciatica. BMJ. (2007) 334:1313–7. doi: 10.1136/bmj.39223.428495.BE PMC1895638 17585160

[22] Ge L Li J Shang WR Xing X Niu JQ Min KL . Non-operative treatments for lumbar disc herniation: an evidence-based practice guideline. Chin J Evid Based Med. (2024) 24:125–48. doi: 10.7507/1672-2531.202309143

[23] Qaseem A Wilt TJ Mclean RM Forciea MA. Noninvasive treatments for acute, subacute, and chronic low back pain: a clinical practice guideline from the American College of Physicians. Ann Intern Med. (2017) 166:514–30. doi: 10.7326/M16-2367 28192789

[24] Shi F Wu Z Li Z Jin J Lin H Liu N . Exploring potential mechanistic aspects of "Biaoben acupoint" acupuncture for lumbar disc herniation using 4D proteomics. Front Neurol. (2025) 16:1630736. doi: 10.3389/fneur.2025.1630736, 40963934 PMC12436106

[25] Wei XY Wang X Shi GX Tu JF Yang JW Ren MM . Acupuncture modulation of chronic neuropathic pain and its association with brain functional properties. J Pain. (2024) 25:104645. doi: 10.1016/j.jpain.2024.104645, 39089662

[26] Samuelly-Leichtag G Eisenberg E Zohar Y Andraous M Eran A Sviri GE . Mechanism underlying painful radiculopathy in patients with lumbar disc herniation. Eur J Pain. (2022) 26:1269–81. doi: 10.1002/ejp.1947, 35357731 PMC10083974

[27] Hirayama J Yamagata M Takahashi K Moriya H. Effect of noxious electrical stimulation of the peroneal nerve on stretch reflex activity of the hamstring muscle in rats: possible implications of neuronal mechanisms in the development of tight hamstrings in lumbar disc herniation. Spine. (2005) 30:1014–8. doi: 10.1097/01.brs.0000160987.66563.ca, 15864152

[28] Huang JH Xie YF Yang JL Yu YJ Wei GX Pei MR . Clinical application of Feng Jun's Shuji acupuncture. J Zhejiang Chin Med Univ. (2021) 45:866–82. doi: 10.16466/j.issn1005-5509.2021.08.009

[29] Hirabayashi K Miyakawa J Satomi K Maruyama T Wakano K. Operative results and postoperative progression of ossification among patients with ossification of cervical posterior longitudinal ligament. Spine. (1981) 6:354–64. doi: 10.1097/00007632-198107000-00005, 6792717

[30] Bijur PE Silver W Gallagher EJ. Reliability of the visual analog scale for measurement of acute pain. Acad Emerg Med. (2001) 8:1153–7. doi: 10.1111/j.1553-2712.2001.tb01132.x, 11733293

[31] Fukui M Chiba K Kawakami M Kikuchi S-I Konno S-I Miyamoto M . Japanese orthopaedic association back pain evaluation questionnaire. Part 3. Validity study and establishment of the measurement scale. J Orthop Sci. (2008) 13:173–9. doi: 10.1007/s00776-008-1213-y, 18528648 PMC2778667

[32] Wang YL. Observation on the therapeutic effect of lumbar disc herniation treated with different acupuncture therapies (in Chinese). Zhongguo Zhen Jiu. (2013) 33:605–8. 24032192

[33] Xu L Li Y Han C Zhang Y Tu T Gao Y . Clinical effects of Lingxian Tongluo capsules combined with Wentong acupuncture method on patients with lumbar disc herniation (in Chinese). Chin Tradit Patent Med. (2024) 46:1524–8. doi: 10.3969/j.issn.1001-1528.2024.05.017

